# Epidemiology and a Predictive Model of Prognosis Index Based on Machine Learning in Primary Breast Lymphoma: Population-Based Study

**DOI:** 10.2196/45455

**Published:** 2023-06-08

**Authors:** Yushuai Yu, Zelin Xu, Tinglei Shao, Kaiyan Huang, Ruiliang Chen, Xiaoqin Yu, Jie Zhang, Hui Han, Chuangui Song

**Affiliations:** 1 Department of Breast Surgery Fujian Medical University Union Hospital Fuzhou China

**Keywords:** primary breast lymphoma, epidemiology, prognosis, machine learning, disparities

## Abstract

**Background:**

Primary breast lymphoma (PBL) is a rare disease whose epidemiological features, treatment principles, and factors used for the patients’ prognosis remain controversial.

**Objective:**

The aim of this study was to explore the epidemiology of PBL and to develop a better model based on machine learning to predict the prognosis for patients with primary breast lymphoma.

**Methods:**

The annual incidence of PBL was extracted from the surveillance, epidemiology, and end results database between 1975 and 2019 to examine disease occurrence trends using Joinpoint software (version 4.9; National Cancer Institute). We enrolled data from 1251 female patients with primary breast lymphoma from the surveillance, epidemiology, and end results database for survival analysis. Univariable and multivariable analyses were performed to explore independent prognostic factors for overall survival and disease-specific survival of patients with primary breast lymphoma. Eight machine learning algorithms were developed to predict the 5-year survival of patients with primary breast lymphoma.

**Results:**

The overall incidence of PBL increased drastically between 1975 and 2004, followed by a significant downward trend in incidence around 2004, with an average annual percent change (AAPC) of −0.8 (95% CI −1.1 to −0.6). Disparities in trends of PBL exist by age and race. The AAPC of the 65 years or older cohort was about 1.2 higher than that for the younger than 65 years cohort. The AAPC of White patients is 0.9 (95% CI 0.0-1.8), while that of Black patients was significantly higher at 2.1 (95% CI −2.5 to 6.9). We also identified that the risk of death from PBL is multifactorial and includes patient factors and treatment factors. Survival analysis revealed that the patients diagnosed between 2007 and 2015 had a significant risk reduction of mortality compared to those diagnosed between 1983 and 1990. The gradient booster model outperforms other models, with 0.752 for sensitivity and 0.817 for area under the curve. The important features established with the gradient booster model were the year of diagnosis, age, histologic type, and primary site, which were the 4 most relevant variables to explain 5-year survival status.

**Conclusions:**

The incidence of PBL started demonstrating a tendency to decrease after 2004, which varied by age and race. In recent years, the prognosis of patients with primary breast lymphoma has been remarkably improved. The gradient booster model had a promising performance. This model can help clinicians identify the early prognosis of patients with primary breast lymphoma and therefore improve the clinical outcome by changing management strategies and patient health care.

## Introduction

Primary breast lymphoma (PBL) is defined as a kind of lymphoma that is only located in the breast, as well as ipsilateral lymph nodes at the time of initial diagnosis [1,2]. It represents no more than 1% of breast malignancies and less than 3% of extranodal lymphomas [3,4]. Nevertheless, the incidence rate of PBL is rising in recent years and deserves attention [5].

Since PBL is a rare disease, its epidemiological characteristics, treatment, and prognosis remain controversial. In particular, the wide variations of its prognoses, which have been reported in different studies, challenged us to evaluate the prognosis of PBL. The 5-year survival rates ranged from 50% to 90% in the previous reports [6-11]. Certainly, the diversity may be due to different case series, a small sample available, different subtypes, clinical stages, treatment methods, and so on. Research is inconclusive about what will affect the outcome of PBL and how much of an effect change can bring. The 5-year survival rate varies according to different clinical stages: 89% for stage I and 50% for stage II [12]. A huge difference exists in the long-term prognosis in recent years, showing improvement with the development of modern therapy [13]. Age has also been reported as an independent prognostic factor, and cases of elderly patients were complicated by more comorbidities that caused a poor prognosis [14,15]. In some studies, chemotherapy and radiotherapy were associated with longer survival, and there was no benefit from mastectomy [2,16]. However, compared with the above factors, histological subtypes play a more important role. Diffuse large B-cell lymphoma (DLBCL) is the most common subtype, which is more aggressive, followed by follicular and mucosa-associated lymphoid tissue (MALT) lymphoma with indolent behavior [13,17]. Picasso et al [18] found that tumors in 50% of patients with primary breast lymphoma were located in the upper outer quadrant and 25% were in the upper inner quadrant; however, there are no studies that try to analyze prognosis between different primary sites. Consequently, the factor of the primary site was also incorporated into our study. There are many other factors that may contribute to the outcome of PBL, but it is not clear about the role of each one.

In order to build up a reliable way to predict the prognosis of patients with primary breast lymphoma, we need to combine all potential prognostic factors with different weight ratios in 1 model. Since it is difficult to set up an effective model in the traditional way under complex interference factors, for example, Nomogram, we use machine learning and the Surveillance, Epidemiology, and End Results (SEER) database to conduct our study. First, we investigated the epidemiology, clinicopathologic features, treatment modalities, and outcomes of PBL. Second, we tried to establish a predictive model with the assistance of machine learning including 11 prognostic factors (age, race, year of diagnosis, marital status, laterality, primary tumor site, histology, Ann Arbor stage, surgery status, radiation status, and chemotherapy status). We believe our work may help with the evaluation of patients with primary breast lymphoma in the future.

## Methods

### Data Source and Study Population

The annual incidence of PBL was extracted from the SEER database between 1975 and 2019 to examine national trends, and all incidence rates were age adjusted. Since the Ann Arbor staging was not available until 1983, patients diagnosed between 1975 and 1982 were not included in the survival analysis and the establishment of the machine learning model. Finally, we enrolled 1251 patients using SEER∗Stat (version 8.3.9; National Cancer Institute) for survival analysis, according to the following inclusion criteria: female, year of diagnosis from 1983 to 2015, the age of diagnosis more than 15 years, breast lymphoma as the only primary malignant cancer diagnosis, and Ann Arbor stage I-II. The exclusion criteria were as follows: Ann Arbor stage III-IV (because these were considered unlikely to be in accordance with extranodal disease) or unknown information, younger than 15 years old, multiple tumors, male cases, and patients who died within 30 days. This study tracked the duration of follow-up starting from the day of diagnosis to December 31, 2019, or the date of death, which can provide follow-up data for more than 5 years. Patient characteristics and treatment courses in our study were identified. The data related to age, race, year of diagnosis, marital status, laterality, primary tumor site, histology, Ann Arbor stage, surgery status, radiation status, and chemotherapy status. Surgery was divided into mastectomy and breast-conserving surgery. We cannot further classify chemotherapy and radiotherapy as the SEER database does not provide detailed chemotherapy and radiotherapy data, such as the regimen, dose, and duration. However, anthracycline-based chemotherapy regimens and radiotherapy of extranodal lesions are the primary treatment options for patients with primary breast lymphoma [2,19,20], so these limitations did not influence our results much.

### Ethics Approval

Our primary data were extracted from the SEER database, which is publicly available. We got permission for data extraction and usage after signing a data-use agreement for the SEER 1975-2019 research data file. Consequently, human subject research ethics review and informed consent were exempted from this study. We confirm that the information of enrolled patients was anonymous or deidentified. In addition, all statistical analyses were conducted in accordance with the regulations of the SEER Program.

### Outcome Measurement

The primary outcome of the study is overall survival (OS). It was defined as from the date of initial diagnosis to the date of death by any cause including PBL. Patients who were alive on the date of the last follow-up were censored. Disease-specific survival (DSS), which served as a secondary study outcome in our study, was defined as from the date of diagnosis to the date of death due to PBL.

### Statistical Analysis

The incidence of PBL for trend analysis was retrieved from the SEER database. The time trends in incidence for PBL were assessed and fit using Joinpoint software (version 4.9; National Cancer Institute) based on log-linear models. Annual percentage change and average annual percent change (AAPC) were calculated to indicate the direction and magnitude of the trends. In order to explore the influences of demographic differences for PBL incidence, the overall population was stratified into different groups, including age and race.

The Kaplan-Meier method was used to generate survival curves. The log-rank test was performed to determine the differences between different demographic and clinical characteristics of PBL patients. Hazard ratio (HR) with 95% CI was identified by using a Cox proportional hazard regression model to determine the factors associated with the outcomes. These statistical analyses were conducted by using SPSS (version 26.0; IBM Corp), and a *P* value of less than .05 was considered as a statistical difference.

**Figure 1:**
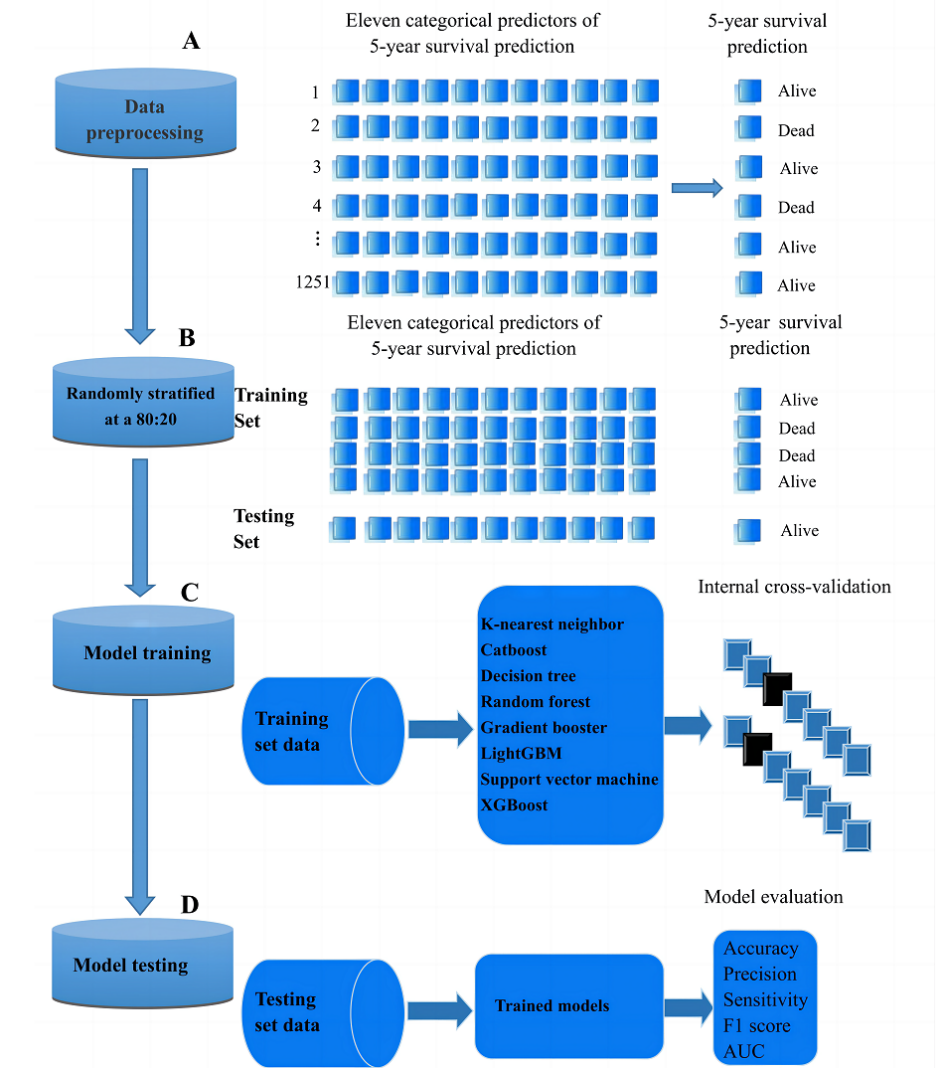
The flowchart of machine learning development process. AUC: area under the curve.

Eleven categorical predictors including age, race, year of diagnosis, marital status, laterality, primary tumor site, histology, Ann Arbor stage, surgery status, radiation status, and chemotherapy status were collected to build a machine learning model for 5-year survival prediction (Figure 1). The package of “MissForest” was used to impute missing values in the data set. Of all enrolled patients, 35.1% (n=439) of patients did not have information on the primary site, 11.2% (n=140) of patients were missing information on the histologic type, and 18.1% (n=226) of patients had no treatment information regarding surgery. The portions of missing values were far less than the cutoff of heavy missingness (75%), which promises good performance of the “MissForest” algorithm [21]. Before building machine learning models, all patients with primary breast lymphoma were randomly divided into a training set and a testing set, at an 80:20 ratio. In our study, 8 machine learning algorithms were used, including K-nearest neighbor, CatBoost, decision tree, random forest method, Gradient Boost, LightGBM, support vector machine, and XGBoost models. A 10-fold internal cross-validation was used to identify the optimal parameters, which provided the highest degree of accuracy in each model. Subsequently, the performance of all machine learning algorithms was evaluated in a testing set, and evaluation measures consisted of accuracy, precision, sensitivity, F1 score, and area under the receiver operating characteristic curve (AUC). The contribution of each element to the machine learning model was evaluated by using feature importance based on the package of “partial_dependence.” Python (version 3.8; Python Software Foundation) was used in these processes.

## Results

### Baseline Characteristics of Patients

The baseline clinical characteristics of the patients are shown in Table 1. Overall, a total of 1251 eligible patients were enrolled in our study. Among the patients, 540 (43.2%) were <65 years and 711 (56.8%) were ≥65 years. A total of 31 (2.5%) patients had tumors in the bilateral, and 1220 (97.5%) patients had tumors in the unilateral. The Ann Arbor stages were distributed as follows: 976 (78%) cases were stage I and 275 (22%) cases were stage II. DLBCL was the most common histologic type of PBL followed by MALT and follicular lymphoma (FL), accounting for 43.4%. In addition, the enrolled patients were not inclined to accept local therapy, including surgery (no surgery vs breast-conserving and mastectomy: n=656, 52.4% vs n=369, 29.5%) and radiotherapy (no radiation vs radiation: n=1031, 82.4% vs n=220, 17.6%), while the percentage of patients in no chemotherapy and chemotherapy was about the same (no chemotherapy vs chemotherapy: n=656, 52.4% vs n=595, 47.6%).

**Table 1:** Baseline characteristics of primary breast lymphoma.

Characteristics		Patients (N=1251), n (%)
**Age (years)**		
	<65	540 (43.2)
	≥65	711 (56.8)
**Race**		
	White	1016 (81.2)
	Black	104 (8.3)
	Other^a^	131 (10.5)
**Marital status**		
	Married	633 (50.6)
	Not married^b^	618 (49.4)
**Laterality**		
	Unilateral	1220 (97.5)
	Bilateral	31 (2.5)
**Year of diagnosis**		
	1983-1990	71 (6)
	1991-1998	166 (13.3)
	1999-2006	401 (32.1)
	2007-2015	613 (49)
**Primary site**		
	Axillary tail	19 (1.5)
	Central portion	74 (6)
	Inner quadrant	77 (6.2)
	Lower-inner quadrant	49 (3.9)
	Lower-outer quadrant	52 (4.2)
	Nipple	9 (1)
	Overlapping lesion	223 (17.8)
	Upper-outer quadrant	309 (24.7)
	NA^c^	439 (35.1)
**Histologic type**		
	DLBCL^d^	543 (43.4)
	MALT^e^	241 (19.3)
	CLL/SLL^f^	58 (4)
	FL^g^	192 (15.3)
	ALCL^h^	27 (2)
	Other^i^	50 (4)
	NA	140 (11.2)
**Ann arbor stage**		
	I	976 (78)
	II	275 (22)
**Surgery approach**		
	No surgery	656 (52.4)
	BCS^j^	333 (26.6)
	Mastectomy	36 (3)
	NA	226 (18.1)
**Radiation status**		
	No	1031 (82.4)
	Yes	220 (17.6)
**Chemotherapy** **status**		
	No	656 (52.4)
	Yes	595 (47.6)

^a^Other includes American Indian/Alaskan Native, Asian/Paciﬁc Islander, and unknown.

^b^Not married includes divorced, separated, single (never married), unmarried or domestic partner, and widowed.

^c^NA: not available.

^d^DLBCL: diffuse large B-cell lymphoma.

^e^MALT: mucosa-associated lymphoid tissue.

^f^CLL/SLL: chronic lymphocytic leukemia/small lymphocytic lymphoma.

^g^FL: follicular lymphoma.

^h^ALCL: anaplastic large cell lymphoma.

^i^Other includes anaplastic large cell lymphoma, angioimmunoblastic T-cell lymphoma, Burkitt lymphoma, extranodal NK-/T-cell lymphoma, nasal type, Mantle cell lymphoma, peripheral T-cell lymphoma, precursor B-lymphoblastic lymphoma, subcutaneous panniculitis-like T-cell lymphoma, T lymphoblastic leukemia/lymphoma.

^j^BCS: breast-conserving surgery.

### Incidence of Breast Lymphoma

**Figure 2:**
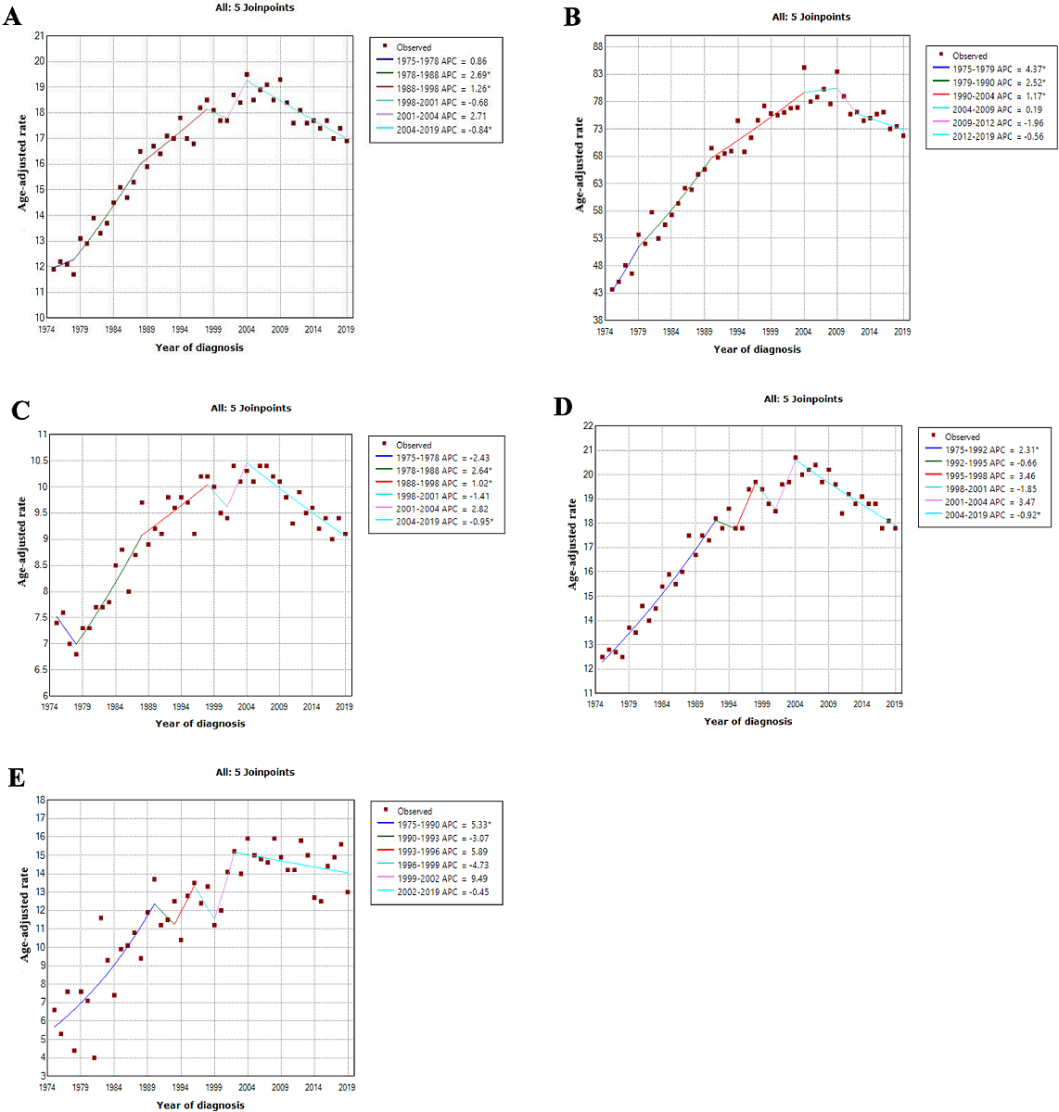
Trend in the incidence of primary breast lymphoma from 1975 to 2019: (A) Overall; (B) Age ≥65 (years); (C) Age <65 (years); (D) White; and (E) Black. *Indicates that the APC is significantly different from 0 at α=.05 level. Final selected model: 2 joinpoints. APC: annual percent change.

The annual percentage change and AAPC for patients with primary breast lymphoma by age and race from 1975 to 2019 are reported in Table 2 and Figure 2. The results demonstrate a remarkable AAPC growth trend of 0.8 (95% CI 0.1-1.5) of patients with primary breast lymphoma during the period 1975 to 2019. The incidence of PBL appears to have a turning point around 2004. From 1975 to 2004, an upward trend was observed, followed by a decline from 2004 to 2019 (AAPC=−0.8; 95% CI −1.1 to −0.6). The AAPC of the ≥65 years cohort was about 1.2 higher than that for the <65 years cohort, which revealed that the incidence of PBL increased slowly with increasing age. The AAPC of White patients is 0.9 (95% CI 0.0-1.8), while that of Black patients was significantly higher at 2.1 (95% CI −2.5 to 6.9). Generally, the PBL incidence substantially increased for the White population between 1975 and 2004 followed by a downward trend for the period between 2004 and 2019. The Black patient cohort also has a similar trend; however, the peak was in 2002 and the incidence has declined noticeably slower than for White patients.

**Table 2:** Trends in age-standardized incidence rates of primary breast lymphoma in 1975-2019.

Model	Age ≥65 (years)		Age <65 (years)		White		Black		Overall	
	Year	APC^a^ (95% CI)	Year	APC (95% CI)	Year	APC (95% CI)	Year	APC (95% CI)	Year	APC (95% CI)
Trend 1	1975-1979	4.4^b^ (0.8 to 8.0)	1975-1978	−2.4 (−7.9 to 3.3)	1975-1992	2.3^b^ (2.0 to 2.6)	1975-1990	5.3^b^ (2.8 to 7.9)	1975-1978	0.9 (−4.0 to 5.9)
Trend 2	1979-1990	2.5^b^ (1.9 to 3.2)	1978-1988	2.6^b^ (1.7 to 3.6)	1992-1995	−0.7 (−7.5 to 6.7)	1990-1993	−3.1 (−34.3 to 43.1)	1978-1988	2.7^b^ (1.9 to 3.5)
Trend 3	1990-2004	1.2^b^ (0.8 to 1.5)	1988-1998	1.0^b^ (0.2 to 1.8)	1995-1998	3.5 (−3.5 to 10.9)	1993-1996	5.9 (−25.8 to 51.1)	1988-1998	1.3^b^ (0.6 to 1.9)
Trend 4	2004-2009	0.2 (−1.8 to 2.2)	1998-2001	−1.4 (−9.4 to 7.3)	1998-2001	−1.9 (−8.3 to 5.0)	1996-1999	−4.7 (−31.6 to 32.7)	1998-2001	−0.7 (−7.0 to 6.1)
Trend 5	2009-2012	−2.0 (−7.9 to 4.3)	2001-2004	2.8 (−5.1 to 11.4)	2001-2004	3.5 (−2.4 to 9.7)	1999-2002	9.5 (−20.0 to 49.9)	2001-2004	2.7 (−3.6 to 9.4)
Trend 6	2012-2019	−0.6 (−1.3 to 0.2)	2004-2019	−1.0^b^ (−1.3 to 0.6)	2004-2019	−0.9^b^ (−1.2 to 0.7)	2002-2019	−0.5 (−1.3 to 0.4)	2004-2019	−0.8^b^ (−1.1 to 0.6)
AAPC^c^	1975-2019	1.2^b^ (0.6 to 1.8)	1975-2019	0.4 (−0.5 to 1.3)	1975-2019	0.9 (0.0 to 1.8)	1975-2019	2.1 (−2.5 to 6.9)	1975-2019	0.8^b^ (0.1 to 1.5)

^a^APC: annual percentage change.

^b^Significantly different from 0 at α=.05 (*P*<.05). There are 5 joinpoints for each model.

^c^AAPC: average annual percent change.

### Survival Analysis

**Figure 3:**
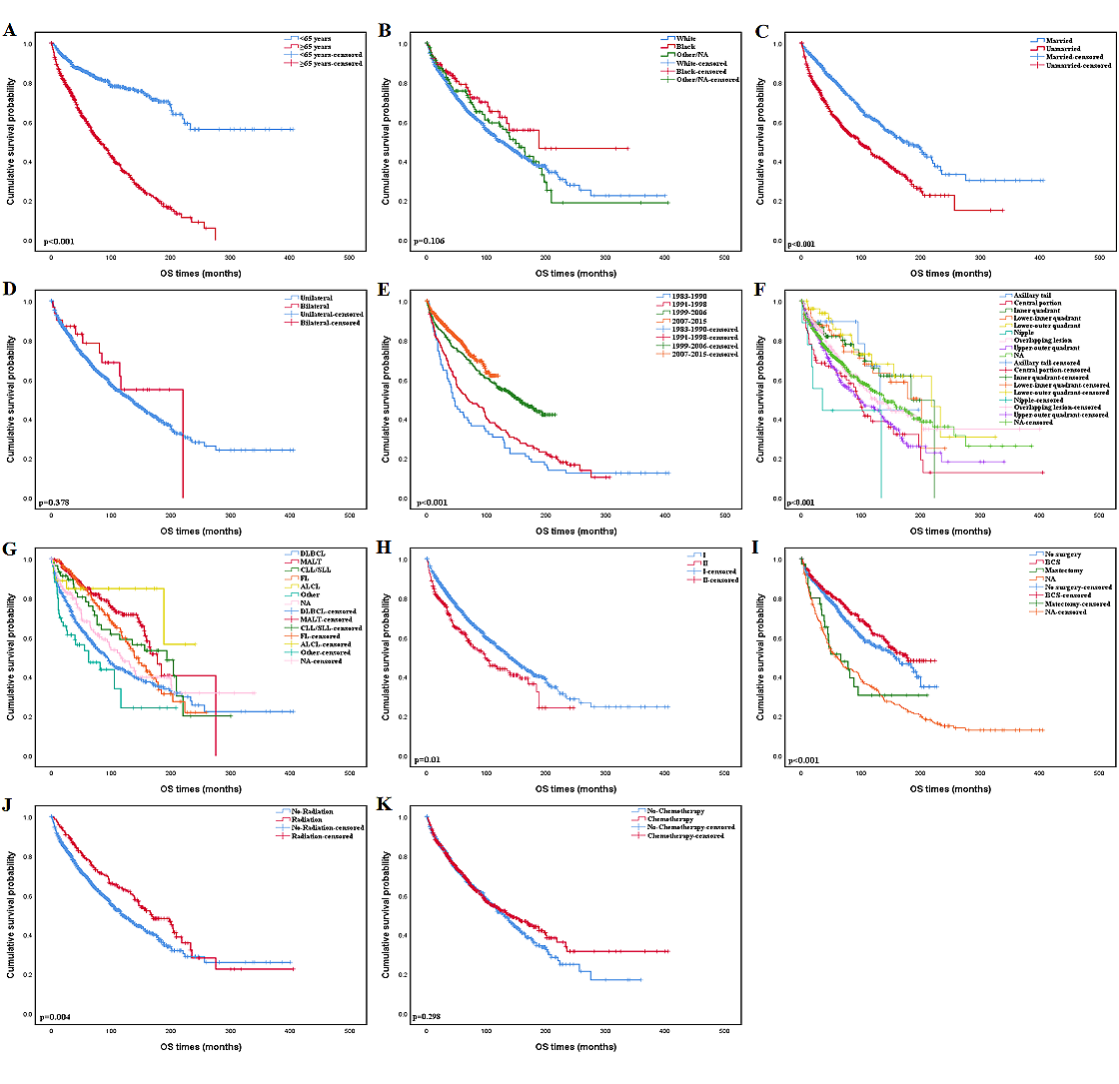
Kaplan-Meier estimate of overall survival by subgroup analysis: (A) age, (B) race, (C) marital status, (D) laterality, (E) year of diagnosis; (F) primary tumor site, (G) histology; (H) Ann Arbor stage, (I) surgery status, (J) radiation status, and (K) chemotherapy status. OS: overall survival.

**Figure 4:**
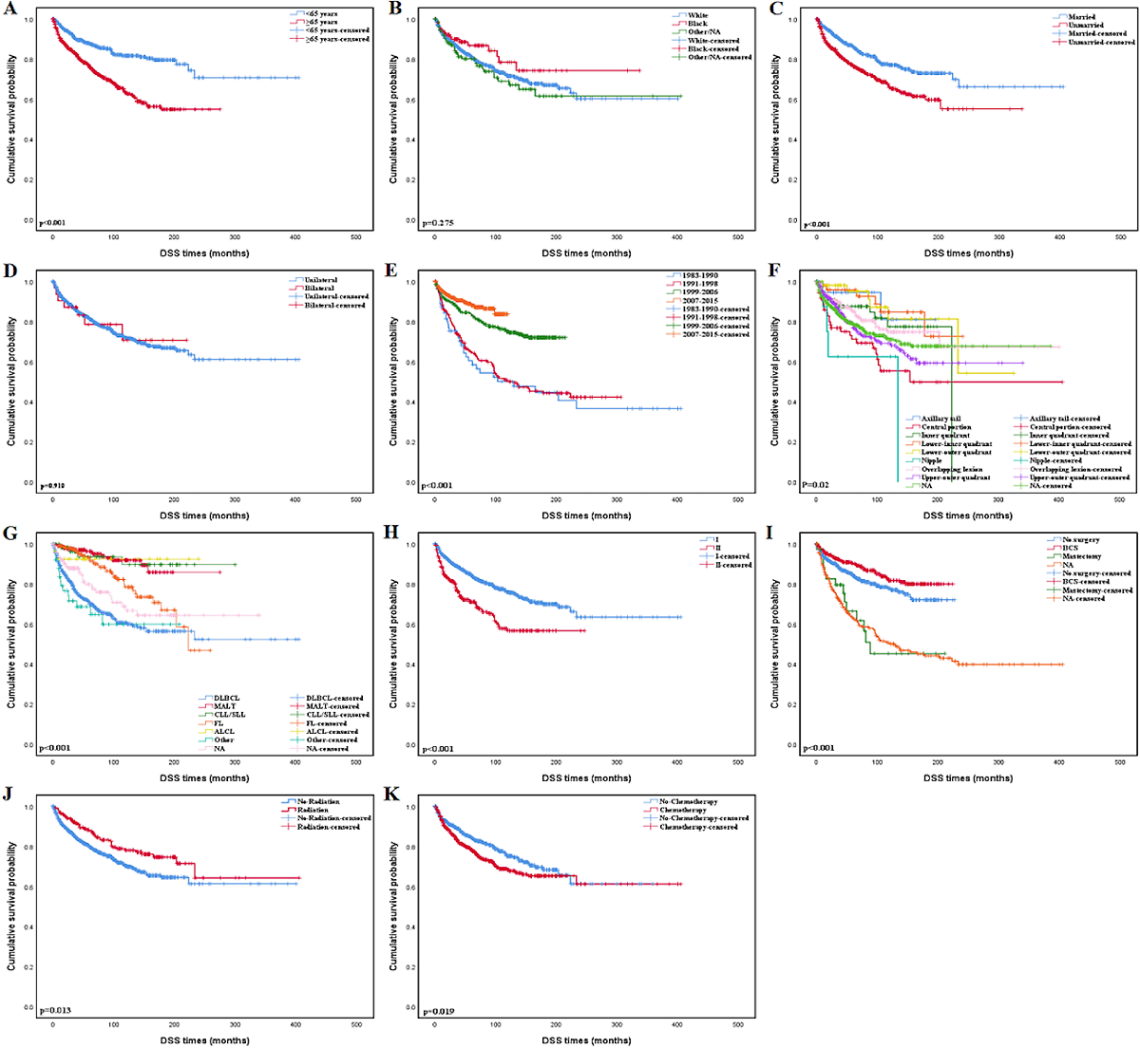
Kaplan-Meier estimate of disease-specific survival by subgroup analysis: (A) age, (B) race, (C) marital status; (D) laterality; (E) year of diagnosis, (F) primary tumor site; (G) histology, (H) Ann Arbor stage, (I) surgery status, (J) radiation status; and (K) chemotherapy status. DSS: disease-specific survival.

The median follow-up time for enrolled patients was 106 months. The Kaplan-Meier curves of OS and DSS based on different baselines in demographic and clinical characteristics are shown in Figures 3 and 4. In our analysis, patients older than 65 years had a noticeably poorer prognosis than younger, which indicated age was an important prognosis factor. The Kaplan-Meier curves for the time period of disease diagnosis are shown in Figure 3E and Figure 4E, the period between 2007 and 2015 was far superior to other time periods. Patients with diffuse large B-cell lymphoma have shorter survival periods compared with other histologic types. Lower Ann Arbor stage (stage I) at diagnosis patients demonstrated a distinctive survival benefit over those with higher Ann Arbor stage (stage II). The primary site in the breast could also influence the prognosis of PBL, as patients with a central portion or nipple neoplasm had a poorer prognosis than patients whose primary tumor sites were in other breast quadrants. The actual laterality of the primary site (bilateral or unilateral) and race did not appear to be related to the prognosis. In terms of therapeutic approaches, breast-conserving surgery and radiotherapy had better OS and DSS.

The univariate Cox regression analysis for each variable is shown in Multimedia Appendix 1. The result of multivariate analysis shown in Table 3 revealed that age, marital status, year of diagnosis, histologic type, Ann Arbor stage, and radiation status were independent prognosis factors. Patients who are older at the time of diagnosis have a higher risk of death than those who are younger (OS: HR 3.458, 95% CI 2.766-4.323, *P*<.001; DSS: HR 1.997, 95% CI 1.511-2.639, *P*<.001, respectively). In terms of marital status, married women had a significant survival advantage (OS: HR 1.549, 95% CI 1.294-1.854, *P*<.001; DSS: HR 1.462, 95% CI 1.140-1.874, *P*=.003, respectively). The patients diagnosed between 2007 and 2015 had a significant risk reduction in mortality than those who were diagnosed between 1983 and 1990 (OS: HR 0.536, 95% CI 0.312-0.919, *P*=.02; DSS: HR 0.411, 95% CI 0.199-0.849, *P*=.02, respectively). Histological type is one of the fundamental features to describe PBL. Patients with mucosa-associated lymphoid tissue, chronic lymphocytic leukemia/small lymphocytic lymphoma (CLL/SLL), and FL had a significant OS and DSS advantage than DLBCL (MALT vs DLBCL: OS, HR 0.396, 95% CI 0.287-0.546, *P*<.001; DSS: HR 0.197, 95% CI 0.111-0.348, *P*<.001; CLL/SLL vs DLBCL: OS, HR 0.448, 95% CI 0.290-0.694, *P*<.001; DSS, HR 0.125, 95% CI 0.045-0.345, *P*<.001; FL vs DLBCL: OS, HR 0.519, 95% CI 0.392-0.686, *P*<.001; DSS, HR 0.396, 95% CI 0.265-0.593, *P*<.001). Patients with a higher Ann Arbor stage (stage II) at diagnosis had a higher hazard of death than those with a lower stage (stage I) at diagnosis, which yielded an HR of 1.414 (95% CI 1.146-1.744) in OS analysis. Radiation lowered the risk of disease-specific mortality and all-cause mortality (OS: HR 0.709, 95% CI 0.551-0.913, *P*=.008; DSS: HR 0.620, 95% CI 0.430-0.893, *P*=.01, respectively). However, no significant difference both in OS and DSS level was detected in patients who received surgery and chemotherapy compared with those who did not (breast-conserving surgery vs no surgery: OS, HR 1.050, 95% CI 0.808-1.364, *P*=.72; DSS, HR 0.948, 95% CI 0.640-1.404, *P*=.79; mastectomy vs no surgery: OS, HR 1.036, 95% CI 0.647-1.658, *P*=.88; DSS, HR 1.196, 95% CI 0.661-2.163, *P*=.55; chemotherapy vs no chemotherapy: OS, HR 0.820, 95% CI 0.661-1.018, *P*=.07; DSS, HR 0.869, 95% CI 0.652-1.159, *P*=.34).

**Table 3:** Multivariate Cox proportional hazard model of disease-specific survival and overall survival in all patients.

Variables		DSS^a^		OS^b^	
		HR^c^ (95% CI)	*P* value	HR (95% CI)	*P* value
**Age (years)**					
	<65	Reference	—^d^	Reference	—
	≥65	1.997 (1.511-2.639)	*<.001* ^e^	3.458 (2.766-4.323)	*<.001*
**Race**					
	White	Reference	—	Reference	—
	Black	0.978 (0.575-1.662)	.93	0.932 (0.632-1.375)	.72
	Other^f^	1.044 (0.720-1.515)	.82	0.999 (0.751-1.329)	>.99
**Marital status**					
	Married	Reference	—	Reference	—
	Not married^g^	1.462 (1.140-1.874)	*.003*	1.549 (1.294-1.854)	*<.001*
**Laterality**					
	Unilateral	Reference	—	Reference	—
	Bilateral	1.179 (0.537-2.592)	.68	0.863 (0.464-1.603)	.64
**Year of diagnosis**					
	1983-1990	Reference	—	Reference	—
	1991-1998	0.975 (0.645-1.474)	.90	0.810 (0.592-1.107)	.19
	1999-2006	0.604 (0.299-1.216)	.16	0.610 (0.361-1.032)	.07
	2007-2015	0.411 (0.199-0.849)	*.02*	0.536 (0.312-0.919)	*.02*
**Primary site**					
	Axillary tail	Reference	—	Reference	—
	Central portion	1.812 (0.425-7.730)	.42	1.371 (0.538-3.496)	.51
	Inner quadrant	1.370 (0.305-6.145)	.68	0.866 (0.325-2.306)	.77
	Lower-inner quadrant	0.927 (0.185-4.642)	.93	1.027 (0.376-2.807)	.96
	Lower-outer quadrant	0.718 (0.143-3.596)	.69	0.770 (0.275-2.157)	.62
	Nipple	2.123 (0.375-12.010)	.40	1.848 (0.547-6.249)	.32
	Overlapping lesion	1.022 (0.244-4.272)	.98	1.018 (0.411-2.525)	.97
	Upper-outer quadrant	1.398 (0.339-5.764)	.64	1.301 (0.529-3.199)	.57
	NA^h^	1.222 (0.298-5.012)	.78	0.995 (0.405-2.441)	.99
**Histologic type**					
	DLBCL^i^	Reference	—	Reference	—
	MALT^j^	0.197 (0.111-0.348)	*<.001*	0.396 (0.287-0.546)	*<.001*
	CLL/SLL^k^	0.125 (0.045-0.345)	*<.001*	0.448 (0.290-0.694)	*<.001*
	FL^l^	0.396 (0.265-0.593)	*<.001*	0.519 (0.392-0.686)	*<.001*
	ALCL^m^	0.234 (0.057-0.967)	*.045*	0.409 (0.165-1.013)	.05
	Other^n^	1.133 (0.673-1.908)	.64	1.357 (0.919-2.005)	.13
	NA	0.556 (0.373-0.830)	*.004*	0.659 (0.492-0.882)	*.005*
**Ann Arbor stage**					
	I	Reference	—	Reference	—
	II	1.847 (1.412-2.416)	*<.001*	1.414 (1.146-1.744)	*.001*
**Surgery approach**					
	No surgery	Reference	—	Reference	—
	BCS^o^	0.948 (0.640-1.404)	.79	1.050 (0.808-1.364)	.72
	Mastectomy	1.196 (0.661-2.163)	.55	1.036 (0.647-1.658)	.88
	NA	1.386 (0.760-2.528)	.29	1.319 (0.839-2.075)	.23
**Radiation status**					
	No	Reference	—	Reference	—
	Yes	0.620 (0.430-0.893)	*.01*	0.709 (0.551-0.913)	*.008*
**Chemotherapy status**					
	No	Reference	—	Reference	—
	Yes	0.869 (0.652-1.159)	.34	0.820 (0.661-1.018)	.07

^a^DSS: disease-specific survival.

^b^OS: overall survival.

^c^HR: hazard ratio.

^d^Not applicable.

^e^Italics indicate statistical significance.

^f^Other includes American Indian/Alaskan native, Asian/Paciﬁc Islander, and unknown.

^g^Not married includes divorced, separated, single (never married), unmarried or domestic partner, and widowed.

^h^NA: not available.

^i^DLBCL: diffuse large B-cell lymphoma.

^j^MALT: mucosa-associated lymphoid tissue.

^k^CLL/SLL: chronic lymphocytic leukemia/small lymphocytic lymphoma.

^l^FL: follicular lymphoma.

^m^ALCL: anaplastic large cell lymphoma.

^n^Other includes anaplastic large cell lymphoma; angioimmunoblastic T-cell lymphoma; Burkitt lymphoma; extranodal NK-/T-cell lymphoma, nasal type; Mantle cell lymphoma; Peripheral T-cell lymphoma; Precursor B-lymphoblastic lymphoma; Subcutaneous panniculitis-like T-cell lymphoma; T lymphoblastic leukemia/lymphoma.

^o^BCS: breast-conserving surgery.

### Machine Learning–Based 5-Year Survival Prediction in Patients With Primary Breast Lymphoma

**Figure 5:**
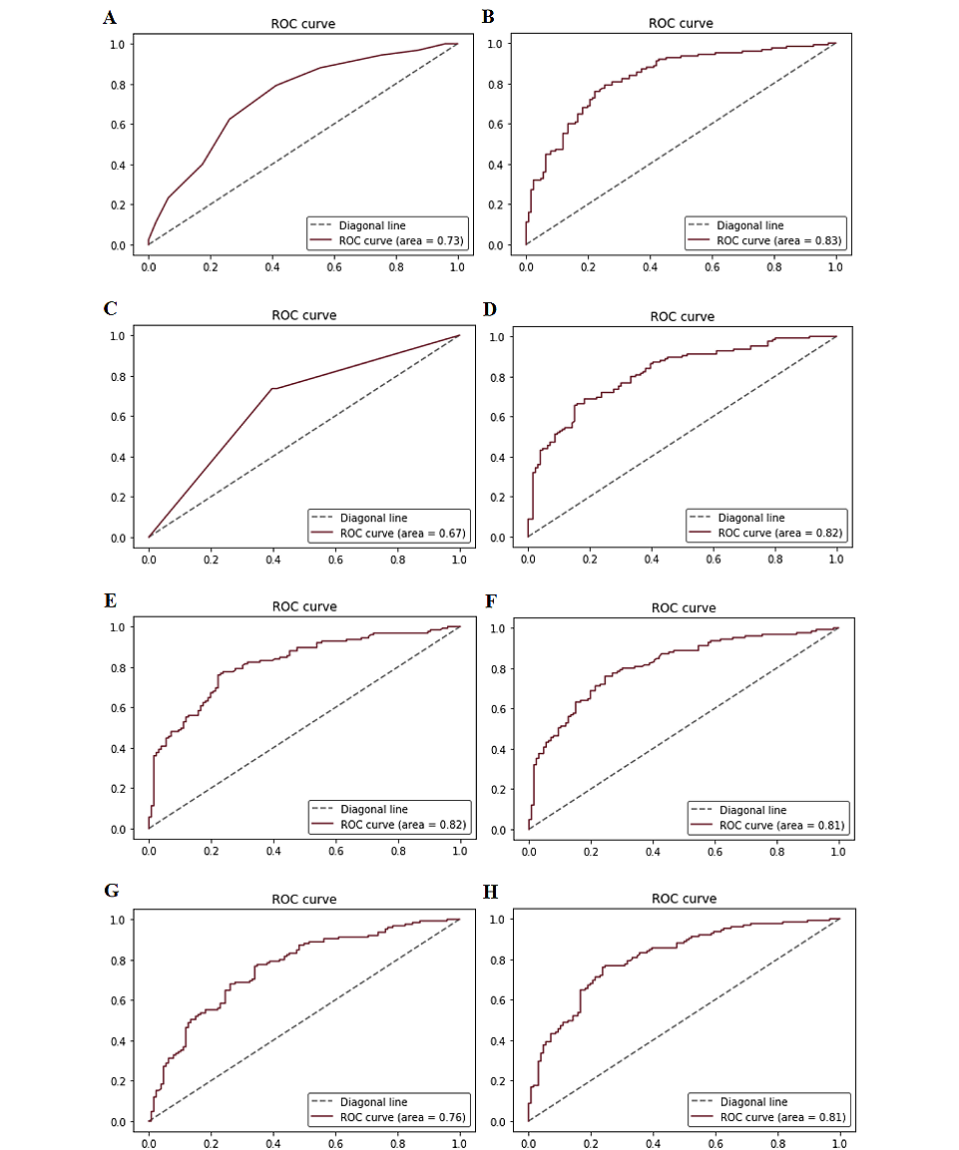
Receiver operating characteristic curves for all models. ROCs for all models: (A) K-nearest neighbor; (B) Catboost; (C) decision tree; (D) random Forest; (E) gradient booster; (F) LightGBM; (G) support vector machine; (H) XGBoost. ROC: receiver operating characteristic curve.

We used a 1251-patient data set for training 8 machine learning models to predict the 5-year survival after PBL diagnosis. The performance of these 8 algorithms is presented in detail in Table 4. The resulting confusion matrix is shown in Multimedia Appendix 2. For the test data set, the sensitivities were K-nearest neighbor model (0.624), Catboost model (0.736), decision tree model (0.736), random forest model (0.720), gradient booster model (0.752), LightGBM model (0.712), support vector machine model (0.696), and XGBoost model (0.728). The AUCs were K-nearest neighbor model (0.735), Catboost model (0.829), decision tree model (0.667), random forest model (0.817), gradient booster model (0.817), LightGBM model (0.814), support vector machine model (0.761), and XGBoost model (0.811). The receiver operating characteristic curves of the 8 models are shown in Figure 5. Due to the design of our study, we focused primarily on testing the sensitivity of patients at high risk of experiencing death in the fifth year. The gradient booster model demonstrated the highest accuracy, precision, sensitivity, and F1 score of all these 8 models; the model also shows a high AUC. Accordingly, the gradient booster algorithm proved to be the most appropriate model for this study. Multimedia Appendix 3 indicates the importance scores for each variable used in the gradient booster, which suggested that year of diagnosis, age, histologic type, and primary site were the 4 most relevant variables to explain 5-year survival status.

**Table 4:** Model performance for the 5-year survival.

Algorithms	Accuracy	Precision	Sensitivity	F1 score	AUC^a^
K-nearest neighbor	0.721	0.703	0.624	0.661	0.735
Catboost	0.757	0.767	0.736	0.751	0.829
Decision tree	0.669	0.648	0.736	0.689	0.667
Random forest	0.721	0.720	0.720	0.720	0.817
Gradient booster	0.765	0.770	0.752	0.761	0.817
LightGBM	0.745	0.761	0.712	0.736	0.814
Support vector machine	0.685	0.680	0.696	0.688	0.761
XGBoost	0.745	0.752	0.728	0.740	0.811

^a^AUC: area under the curve.

## Discussion

### Principal Findings

To understand the changes in the incidence trend and survival of patients with primary breast lymphoma over the last 40 years, we conducted a large population-based epidemiological study with data from the SEER database. The results of our study showed the overall incidence of PBL appeared to start a downward trend around 2004. Our study is the first one to report this encouraging phenomenon. We also developed and validated novel models based on machine learning algorithms for predicting 5-year survival. In particular, the gradient booster model achieved the most promising results in terms of AUC, accuracy, precision, sensitivity, and F1 score.

PBL is a comparatively rare form of extranodal lymphoma, and global reports about the incidence of PBL remain limited. According to the literature from 1975 to 2000, the overall incidence of PBL had increased dramatically but has lately stabilized [13]. Our results indicate a similar trend. By extracting data from 1975 to 2019 from the SEER database, we identified an overall increase in the incidence of PBL during 1975-2004, with a peak incidence rate in 2004. In comparison, a previous article that estimated the non-Hodgkin lymphoma (NHL) incidence for 185 countries in 2018 using the IARC’s GLOBOCAN database also showed an increasing trend in the incidence of PBL until the 1990s and the stabilization or decrease thereafter [22]. Genetic determinants, lifestyle, and environmental factors have been considered as causes for NHL [23]. Infections with hepatitis C virus, Epstein-Barr virus, *Helicobacter pylori*, and HIV increase the risk of NHL, and the reduced incidence of lymphoma can be partly explained by the decrease in the incidence of virus infections and advances in antiretroviral therapy [24-26]. In our study, we focused on the possible impact of age and race on the incidence of disease. Our data show that the AAPC in an older population was 3 times higher than in a younger population. Consistent with our results, a report from Cancer Research UK indicated that a high proportion of NHL diagnoses occurred in older people, with the highest incidence in people aged 80 to 84 years [27]. Our data revealed that the incidence of PBL increased slowly with increasing age. In addition, race was also significantly associated with the incidence rate, as the AAPC of Black people was much higher than that of White people. The apparent differences in the incidence of PBL by race may be related to the levels of access to health care, availability of diagnostic services, endemic infections, and environmental factors [28]. In summary, this preliminary finding suggests that future biological and epidemiological analyses on PBL should be stratified by age and ethnic background.

Building up a reliable way to predict the prognosis of patients with primary breast lymphoma plays a crucial role in the early determination of the treatment of patients with primary breast lymphoma. Currently, we determine the prognosis for patients with primary breast lymphoma mainly based on clinicopathological characteristics; however, the limited data cannot provide adequate information for clinicians to deal with this extremely complex disease. Although a study developed a nomogram to predict the survival of PBL, Ann Arbor stage III and IV patients were all involved in their study, which may affect the effectiveness of the model [1,2,15]. Additionally, treatment approaches that have a critical impact on the prognosis of the disease including surgery, chemotherapy, and radiotherapy were not incorporated into the model due to the lack of statistical significance [15]. These omissions make any conclusions highly controversial. Machine learning is being widely used in the medical field for disease diagnosis, prognosis, therapeutic modality selection, and so on [29-31]. A machine learning model can automatically adjust the weight of the factors to make the best use of the data. Our study used the 5-year survival of patients with primary breast lymphoma as the predictive end point, an important point for early determination of prognosis. The results showed that the performance of the gradient booster model was superior to that of all the other models and is regarded as a promising model. Machine learning techniques have also been used to predict the 5- and 10-year recurrence of invasive breast cancer. Massafra et al [32] enrolled 529 patients with breast cancer from Italy, reaching good AUC values of 0.771 and 0.763 for the recurrence prediction at 5 and 10 years. There are 28 features associated with primary breast cancer clinicopathological characteristics and treatment programs that were used to train models, which are more detailed than our cohort. Twenty-eight features were used because breast cancer can provide more clinicopathological characteristics and have more treatment methods than PBL. However, our established gradient booster model shows a higher AUC value, which reflects that it is still possible to train promising prediction models, even with limited predictors.

The important features established with the gradient booster model were the year of diagnosis, age, histologic type, and primary site as the 4 most relevant variables to explain the 5-year survival status. The year of diagnosis was considered as the most meaningful predictive prognostic factor, which suggested that current treatments, probably in combination with newer systemic treatments (likely rituximab), have improved the control of this disease [13]. Rituximab was approved by the US Food and Drug Administration for marketing in the United States on November 26, 1997, and was a milestone for the treatment of PBL. This may be an important factor in why our results demonstrate a significant improvement in the prognosis of patients with primary breast lymphoma after 1999. Age and histologic type were confirmed as important prognosis factors in the machine learning model, like other investigations [3,15,33,34]. Undoubtedly, elderly PBL patients become complicated by more comorbidities and poor drug tolerance or physical condition, which may have a direct negative impact on survival time [35,36]. In accordance with the past research, different histologic types demonstrated dramatic survival differences, which can be partly due to some cell phenotypes with a high proliferation feature and association with a poor treatment response [33,34,37]. Interestingly, the primary site of PBL is thought to be an important predictive prognostic feature in the gradient booster model, which was never reported or analyzed by other series. There are significant differences in the primary site of PBL, a cohort found PBL predisposes to locate in the upper outer quadrant, which is also confirmed in our study [18]. Our Kaplan-Meier survival curves across different primary sites suggested that the central portion and nipple site have worse outcomes. This may be explained by the anatomy of breast lymphatic drainage. The subareolar plexus collects lymphatics originating from breast parenchyma, and then they drain to the lymph nodes of the axilla. Additionally, the deep lymphatic channels connect to the superficial cutaneous lymphatic plexus, especially in the subareolar plexus around the nipple [38,39]. The primary site of central portion or nipple may be prone to be present with lymphatic vessel invasion, causing a poor prognosis; however, this needs further study in future trials or experimental research.

Our study provides information on the incidence and prognostic factors over the last 40 years involving a sufficient sample size. In addition, the first prognostic model for patients with primary breast lymphoma based on a machine learning algorithm was performed for clinical use. We are confident that we have built a predictive model with a good performance, and it can provide physicians with an easy-to-access predictive tool and facilitate a more personalized follow-up strategy, management strategies, and patient care for patients with primary breast lymphoma. The model may help to identify patients who are at a higher risk of a poor outcome and will require more aggressive treatment. However, there are some limitations to our study. Due to limited information from the SEER database, we did not include variables such as biomarkers, chemotherapy regimen, radiotherapy dosing, and targeted drugs in our model. Therefore, when interpreting the results, caution should be used. Further, the database may not capture all relevant patient outcomes including the frequency of central nervous system recurrence, which might potentially affect the interpretation of our results. In addition, the models generated in this study have not been verified in an external validation cohort. In order to achieve this objective, we are collecting related case information to establish a database.

### Conclusions

The incidence of PBL started demonstrating a tendency to decrease after 2004, which varied by age and race. In recent years, the prognosis of PBL has been remarkably improved. The gradient booster model had a promising performance. This model can help clinicians identify the prognosis of patients with primary breast lymphoma early and therefore improve clinical outcomes by changing management strategies and patient health care.

## Appendix

Multimedia Appendix 1 Univariate Cox proportional hazard model of disease-specific survival and overall survival in all patients.

Multimedia Appendix 2 Confusion matrix of 8 algorithms for the 5-year survival status.

Multimedia Appendix 3 Feature importance of the gradient booster model.

## Abbreviations

AAPC: average annual percent change
AUC: area under the curve
DLBCL: diffuse large B-cell lymphoma
DSS: disease-specific survival
HR: hazard ratio
MALT: mucosa-associated lymphoid tissue
NHL: non-Hodgkin lymphoma
OS: overall survival
PBL: primary breast lymphoma
SEER: Surveillance, Epidemiology, and End Results

## Data Availability

The data sets generated and analyzed during this study are available in the Surveillance, Epidemiology, and End Results (SEER) database [40].
