# Supplementary material for: Epidemiology and a Predictive Model of Prognosis Index Based on Machine Learning in Primary Breast Lymphoma: Population-Based Study
Source: JMIR Public Health Surveill. 2023 Jun 8;9:e45455. doi: 10.2196/45455 (PMC10288347; doi:10.2196/45455)
Supplement: Multimedia Appendix 1 [file publichealth_v9i1e45455_app1.docx]

**Multimedia Appendix 1:** Univariate Cox proportional hazard model of disease-specific survival and overall survival in all patients.

| Variables | DSS^a^ | |  | OS^b^ | |
| --- | --- | --- | --- | --- | --- |
|  | HR^c^ (95% CI) | *P* |  | HR^c^ (95% CI) | *P* |
| **Age (years)** |  |  |  |  |  |
| <65 | Reference |  |  | Reference |  |
| ≥65 | 2.256 (1.739-2.926) | **<.001** |  | 3.921 (3.179-4.838) | **<.001** |
| **Race** |  |  |  |  |  |
| White | Reference |  |  | Reference |  |
| Black | 0.708 (0.426-1.175) | .181 |  | 0.677 (0.468-0.979) | **.038** |
| Other^d^ | 1.147 (0.799-1.645) | .457 |  | 0.929 (0.704-1.227) | .604 |
| **Marital status** |  |  |  |  |  |
| Married | Reference |  |  | Reference |  |
| Not married^e^ | 1.684 (1.325-2.141) | **<.001** |  | 1.836 (1.544-2.183) | **<.001** |
| **Laterality** |  |  |  |  |  |
| Unilateral | Reference |  |  | Reference |  |
| Bilateral | 0.958 (0.452-2.029) | .910 |  | 0.765 (0.421-1.391) | .380 |
| **Year of diagnosis** |  |  |  |  |  |
| 1983-1990 | Reference |  |  | Reference |  |
| 1991-1998 | 0.920 (0.616-1.375) | .685 |  | 0.845 (0.625-1.142) | .273 |
| 1999-2006 | 0.403 (0.272-0.597) | **<.001** |  | 0.473 (0.354-0.631) | **<.001** |
| 2007-2015 | 0.270 (0.177-0.410) | **<.001** |  | 0.371 (0.272-0.507) | **<.001** |
| **Primary Site** |  |  |  |  |  |
| Axillary tail | Reference |  |  | Reference |  |
| Central portion | 3.553 (0.843-14.970) | .084 |  | 2.281 (0.904-5.761) | .081 |
| Inner quadrant | 1.556 (0.351-6.893) | .561 |  | 1.043 (0.395-2.755) | .932 |
| Lower-inner quadrant | 1.006 (0.203-4.987) | .994 |  | 1.075 (0.397-2.915) | .887 |
| Lower-outer quadrant | 0.984 (0.198-4.875) | .984 |  | 0.867 (0.312-2.409) | .785 |
| Nipple | 5.218 (0.956-28.492) | .056 |  | 3.252 (0.992-10.659) | .051 |
| Overlapping lesion | 1.704 (0.412-7.047) | .462 |  | 1.510 (0.614-3.714) | .369 |
| Upper-outer quadrant | 2.440 (0.599-9.943) | .213 |  | 2.008 (0.824-4.896) | .125 |
| NA^f^ | 2.253 (0.556-9.129) | .255 |  | 1.521 (0.626-3.700) | .355 |
| **Histologic Type** |  |  |  |  |  |
| DLBCL^g^ | Reference |  |  | Reference |  |
| MALT^h^ | 0.168 (0.099-0.285) | **<.001** |  | 0.419 (0.316-0.557) | **<.001** |
| CLL/SLL^i^ | 0.171 (0.064-0.462) | **<.001** |  | 0.622 (0.413-0.938) | **.024** |
| FL^j^ | 0.426 (0.293-0.618) | **<.001** |  | 0.632 (0.492-0.812) | **<.001** |
| ALCL^k^ | 0.210 (0.052-0.846) | **<.028** |  | 0.318 (0.131-0.769) | **.011** |
| Other^l^ | 1.222 (0.732-2.040) | .444 |  | 1.474 (1.005-2.162) | **.047** |
| NA^f^ | 0.676 (0.464-0.986) | **.042** |  | 0.802 (0.609-1.057) | .118 |
| **Ann Arbor Stage** |  |  |  |  |  |
| I | Reference |  |  | Reference |  |
| II | 1.931 (1.497-2.491) | **<.001** |  | 1.395 (1.142-1.703) | **.001** |
| **Surgery Approach** |  |  |  |  |  |
| No surgery | Reference |  |  | Reference |  |
| BCS^m^ | 0.686 (0.486-0.967) | **.032** |  | 0.840 (0.670-1.053) | .130 |
| Mastectomy | 2.435 (1.396-4.250) | **.002** |  | 1.866 (1.192-2.923) | **.006** |
| NA^f^ | 2.546 (1.941-3.339) | **<.001** |  | 1.992 (1.624-2.443) | **<.001** |
| **Radiation Status** |  |  |  |  |  |
| No | Reference |  |  | Reference |  |
| Yes | 0.665 (0.481-0.919) | **.014** |  | 0.722 (0.579-0.900) | **.004** |
| **Chemotherapy Status** |  |  |  |  |  |
| No | Reference |  |  | Reference |  |
| Yes | 1.327 (1.046-1.683) | **.020** |  | 0.914 (0.771-1.083) | .300 |

^a^DSS: disease-specific survival.

^b^OS: overall survival.

^c^HR: hazard ratio.

^d^Other includes American Indian/Alaskan native, Asian/Paciﬁc Islander, and unknown.

^e^Not married includes divorced, separated, single (never married), unmarried or domestic partner, and widowed.

^f^NA: not available.

^g^DLBCL: diffuse large B-cell lymphoma.

^h^MALT: mucosa-associated lymphoid tissue.

^i^CLL/SLL: chronic lymphocytic leukemia/small lymphocytic lymphoma.

^j^FL: follicular lymphoma.

^k^ALCL: anaplastic large cell lymphoma.

^l^Other includes anaplastic large cell lymphoma; angioimmunoblastic T-cell lymphoma; Burkitt lymphoma; Extranodal NK-/T-cell lymphoma, nasal type; Mantle cell lymphoma; Peripheral T-cell lymphoma; Precursor B-lymphoblastic lymphoma; Subcutaneous panniculitis-like T-cell lymphoma; T lymphoblastic leukemia/lymphoma.

^m^BCS: breast-conserving surgery.

*Bold type indicates statistical significance.*
