# Supplementary material for: Epidemiology and a Predictive Model of Prognosis Index Based on Machine Learning in Primary Breast Lymphoma: Population-Based Study
Source: JMIR Public Health Surveill. 2023 Jun 8;9:e45455. doi: 10.2196/45455 (PMC10288347; doi:10.2196/45455)
Supplement: Multimedia Appendix 2 [file publichealth_v9i1e45455_app2.docx]

**Multimedia Appendix 2:** Confusion matrix of eight algorithms for the five-year survival status.

| Algorithms |  | Predictions |  |
| --- | --- | --- | --- |
|  | True Lable | Dead | Alive |
| K-nearest neighbor | Dead | 93 | 33 |
|  | Alive | 47 | 78 |
| Catboost | Dead | 98 | 28 |
|  | Alive | 33 | 92 |
| Decision tree | Dead | 76 | 50 |
|  | Alive | 33 | 92 |
| Random forest | Dead | 91 | 35 |
|  | Alive | 35 | 90 |
| Gradient booster | Dead | 98 | 28 |
|  | Alive | 31 | 94 |
| LightGBM | Dead | 98 | 28 |
|  | Alive | 36 | 89 |
| Support vector machine | Dead | 85 | 41 |
|  | Alive | 38 | 87 |
| XGBoost | Dead | 96 | 30 |
|  | Alive | 34 | 91 |
