# Supplementary figures and images for: Epidemiology and a Predictive Model of Prognosis Index Based on Machine Learning in Primary Breast Lymphoma: Population-Based Study
Source: JMIR Public Health Surveill. 2023 Jun 8;9:e45455. doi: 10.2196/45455 (PMC10288347; doi:10.2196/45455)

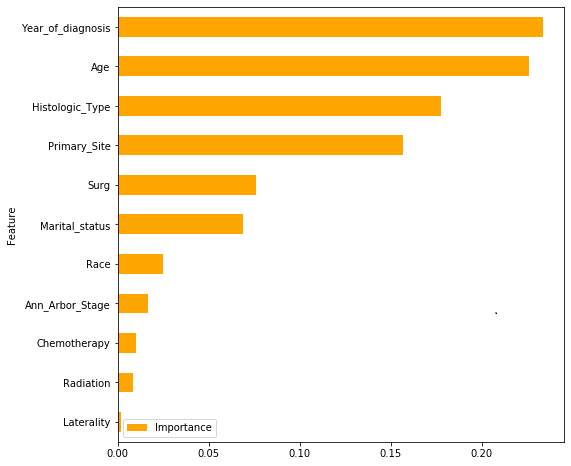

Supplement: Multimedia Appendix 3 [file publichealth_v9i1e45455_app3.png]
